# Marine Bivalve Mollusks As Possible Indicators of Multidrug-Resistant *Escherichia coli* and Other Species of the Enterobacteriaceae Family

**DOI:** 10.3389/fmicb.2017.00024

**Published:** 2017-01-18

**Authors:** Didrik H. Grevskott, Cecilie S. Svanevik, Marianne Sunde, Astrid L. Wester, Bjørn T. Lunestad

**Affiliations:** ^1^National Institute of Nutrition and Seafood ResearchBergen, Norway; ^2^Norwegian Veterinary InstituteOslo, Norway; ^3^Norwegian Institute of Public HealthOslo, Norway

**Keywords:** bivalve mollusks, Enterobacteriaceae, *Escherichia coli*, antibacterial resistance, horizontal gene transfer

## Abstract

The mechanisms for the development and spread of antibacterial resistance (ABR) in bacteria residing in environmental compartments, including the marine environment, are far from understood. The objective of this study was to examine the ABR rates in *Escherichia coli* and other Enterobacteriaceae isolates obtained from marine bivalve mollusks collected along the Norwegian coast during a period from October 2014 to November 2015. A total of 549 bivalve samples were examined by a five times three tube most probable number method for enumeration of *E. coli* in bivalves resulting in 199 isolates from the positive samples. These isolates were identified by biochemical reactions and matrix Assisted Laser Desorption Ionization-Time of Flight Mass Spectrometry, showing that 90% were *E. coli*, while the remaining were species within the genera *Klebsiella, Citrobacter*, and *Enterobacter*. All 199 isolates recovered were susceptibility tested following the European Committee on Antimicrobial Susceptibility Testing disk diffusion method. In total, 75 of 199 (38%) isolates showed resistance to at least one antibacterial agent, while multidrug-resistance were seen in 9 (5%) isolates. One isolate conferred resistance toward 15 antibacterial agents. Among the 75 resistant isolates, resistance toward extended-spectrum penicillins (83%), aminoglycosides (16%), trimethoprim (13%), sulfonamides (11%), tetracyclines (8%), third-generation cephalosporins (7%), amphenicols (5%), nitrofurans (5%), and quinolones (5%), were observed. Whole-genome sequencing on a selection of 10 *E. coli* isolates identified the genes responsible for resistance, including *bla*_CTX-M_ genes. To indicate the potential for horizontal gene transfer, conjugation experiments were performed on the same selected isolates. Conjugative transfer of resistance was observed for six of the 10 *E. coli* isolates. In order to compare *E. coli* isolates from bivalves with clinical strains, multiple-locus variable number tandem repeats analysis (MLVA) was applied on a selection of 30 resistant *E. coli* isolates. The MLVA-profiles were associated with community-acquired *E. coli* strains causing bacteremia. Our study indicates that bivalves represent an important tool for monitoring antibacterial resistant *E. coli* and other members of the Enterobacteriaceae family in the coastal environment.

## Introduction

The development of antibacterial resistance (ABR) is a natural process and ancient among bacteria (Aminov and Mackie, 2007; D’Costa et al., 2011). However, the current global use of antibacterial agents in human and veterinary medicine, as well as in agriculture, are a driving force for ABR development and also increase the release of these substances to the environment (Davies and Davies, 2010).

The intestines of humans and other homeothermic animals are colonized by a dense and diverse microbiota belonging to, among others, the Enterobacteriaceae family (Tancrède, 1992; Dethlefsen et al., 2006). The predominant genus within this family is *Escherichia*, with *Escherichia coli* being the main species. *E. coli* occurs naturally in the large intestine of humans, birds, and terrestrial and marine mammals (Welch, 2006). Most *E. coli* of the large intestine of humans and other homeothermic animals are commensal strains, however opportunistic and pathogenic strains may be present (Strockbine et al., 2015). *E. coli* cause morbidity and mortality as a result of common infections, including enteritis, meningitis, urinary tract, or bloodstream infections (Strockbine et al., 2015). The main sources of infections with pathogenic *E. coli* are consumption of contaminated water and food, as well as through animal contact (ILSI, 2011).

Antibacterial treatments are known to substantially affect the normal intestinal microbiota favoring resistant strains (Sommer and Dantas, 2011). The prevalence of resistant *E. coli* and other bacteria in the intestinal microbiota of humans are shown to be strongly correlated with the use of antibacterial agents (Murray et al., 1982; Bruinsma et al., 2003; van der Veen et al., 2009).

The microbiological communities in coastal environments can be influenced by sewage and runoff from land, concomitantly containing both fecal bacteria as well as residues of antibacterial substances (Martinez, 2009; Alves et al., 2014; Balière et al., 2015). A significant proportion of the antimicrobial agents are excreted unchanged and in a biologically active form (Dolliver and Gupta, 2008; Gillings, 2013; Michael et al., 2013). During periods with heavy rainfall, increased amount of fecal material from land living animals will reach the sea. In addition, high precipitation could cause an overload and possible leakage from sewage systems. Sewage and manure harbor bacteria of high diversity, have a high concentration of organic substances, as well as anthropogenic pollution as heavy metals and antimicrobial agents, which in combination can favor bacterial growth and promote spread of genetic elements through horizontal gene transfer (Moura et al., 2010; Heuer et al., 2011). Bacteria conferring ABR colonizing the intestines of humans and other homeothermic animals, may contribute to the dissemination of antibiotic resistant bacteria (ABR-B) via sewage to the marine environment (Poeta et al., 2005; Penders et al., 2013). The survival of these bacteria in aquatic environments are affected by both abiotic and biotic factors, e.g., nutrient availability, osmotic stress, variations in temperature and pH, and predation (Barcina et al., 1997; Rozen and Belkin, 2001; Campos et al., 2013). Importantly, *E. coli* have the ability to persist in the aquatic habitat due to its genetic flexibility (van Elsas et al., 2011).

The presence of Enterobacteriaceae conferring resistance to antibacterial agents in coastal waters may represent a human health issue, especially in areas used for marine food production or recreational activities (Murugaiyan et al., 2015). Multidrug-resistant (MDR) bacteria have been detected in coastal waters, and could result in the transmission of resistance among marine and contaminating bacteria via exchange of genetic elements, such as plasmids (Wright, 2010; Alves et al., 2014; Moura et al., 2014).

Bivalve mollusks are invertebrates that have an external two-part hinged shell that contains the soft parts. Typical bivalve mollusks comprise among others clams, oysters, mussels, and scallops. As these mollusks are suspension feeders, they actively filter, retain, and concentrates particles from their surrounding water, including free living or particle-bound bacteria (Bernard, 1989; Leff et al., 1992; Maugeri et al., 2004). Bivalve associated members of the Enterobacteriaceae family, may originate from humans and other homeothermic animals either via sewage, by runoff from land, or from representatives of the wild fauna such as birds or marine mammals (Bogomolni et al., 2008). These bivalves are therefore excellent indicators for fecal contamination and will reflect the load of *E. coli* and other bacteria in the Enterobacteriaceae family present in the water column at a given location. However, different environmental conditions, e.g., temperature, water flow rate, and food availability, can affect the filtration rate, consequently also the accumulation of fecal bacteria (Šolić et al., 1999; Strohmeier et al., 2012; Campos et al., 2013).

Bivalve mollusks are good candidate for studies on resistance in bacteria originating from several sources including humans and animals, and gives the possibility of comparing temporal and spatial changes and the potential for exposure to humans by consumption of marine bivalves. The main objective of this study was to examine the ABR rates in Enterobacteriaceae isolates obtained from marine bivalve mollusks collected along the Norwegian coast. In addition, an assessment of the transferability of certain resistance genes, as well as comparing bivalve isolates with clinical isolates of human origin, was performed.

## Materials and Methods

### Sampling and Identification of Bacterial Isolates

As part of the mandatory EU surveillance program (854/2004/EC, 2004) conducted by the Norwegian Food Safety Authority (NFSA), sampling of bivalve mollusks were performed from 57 localities covering the Norwegian coast on several occasions from October 2014 to November 2015. A standardized most probable number (MPN) reference method for enumeration of *E. coli* in bivalves (Oblinger and Koburger, 1975), with Minerals Modified Glutamate Broth (MMGB) (Oxoid, UK) as growth media in combination with verification on Tryptone Bile with X-glucuronide (TBX) agar (Oxoid, UK) (Donovan et al., 1998), was performed as described in Grevskott et al. (2016). A total of 549 bivalves were collected and examined at the National Institute of Nutrition and Seafood Research and the Norwegian Institute of Public Health, as presented in Grevskott et al. (2016). More than a half of the bivalve samples (51%) was harvested from commercially active rearing localities, while the rest were collected from positions established by NFSA for long time reference monitoring purposes of shellfish safety. A total number of 199 bacterial isolates, one from each randomly selected culture-positive bivalve sample (*n* = 335), was grown into pure culture for further analysis.

### Antibacterial Susceptibility Testing

The bacterial isolates were susceptibility tested by disk diffusion on Mueller-Hinton (MH) agar (Oxoid, UK) according to the European Committee on Antimicrobial Susceptibility Testing (EUCAST) (Matuschek et al., 2014). Each bacterial isolate was tested for 24 antibacterial agents, representing 10 drug classes (WHOCC Server, 2016). The following disks (Oxoid, UK) were applied: ampicillin (10 μg), amoxicillin (10 μg), amoxicillin/clavulanic acid (2/1 μg), mecillinam (10 μg), piperacillin/tazobactam (30/6 μg), chloramphenicol (30 μg), ciprofloxacin (5 μg), levofloxacin (5 μg), nalidixic acid (30 μg), norfloxacin (10 μg), nitrofurantoin (100 μg), gentamicin (10 μg), tobramycin (10 μg), streptomycin (25 μg), kanamycin (30 μg), trimethoprim (5 μg), trimethoprim/sulfamethoxazole (1.25/23.75 μg), cefotaxime (5 μg), ceftazidime (10 μg), doxycycline (30 μg), tetracycline (30 μg), colistin sulfate (25 μg), imipenem (10 μg), and meropenem (10 μg). To monitor the quality for each new batch of MH agar, and antibacterial disks, *E. coli* CCUG 17620 was included on a regular basis. The inhibition zones were interpreted according to the EUCAST clinical breakpoint tables v.6.0 (EUCAST, 2016). For some substances breakpoints were not available and for these substances clinical breakpoints given by Clinical and Laboratory Standards Institute (CLSI, 2014) or Indian Council of Medical Research (ICMR, 2009), were used.

### Whole-Genome Sequencing

A selection of 10 isolates was subjected to whole-genome sequencing (WGS). The isolates were selected on the basis of phenotypes showing resistance toward multiple antibacterial agents and/or expressing resistance to critically important agents, such as to third-generation cephalosporins. DNA was isolated by the use of the MagNA Pure 96 DNA and Viral NA Small Volume Kit and a MagNApure 96 instrument (Roche Diagnostics, Germany). The sequencing libraries were prepared using the Kapa HyperPLus Library Preparation Kit (Kapa Biosystems, USA). The isolates were sequenced on an Illumina MiSeq platform (Illumina, USA), producing (2 bp × 250 bp) paired-end reads. The data were adaptor and quality trimmed using Trimmomatic (Bolger et al., 2014), and assembled using SPAdes (Bankevich et al., 2012). The processed sequence data were analyzed for genes encoding resistance to antimicrobial resistance using the web-based ResFinder tool (Zankari et al., 2012), for serotype using the SerotypeFinder tool (Joensen et al., 2015) and for multi-locus sequence types (MLSTs) using the MLSTs tool (Larsen et al., 2012) from Centre for Genomic Epidemiology^1^, at the Technical University of Denmark.

### Conjugation Experiments

The whole-genome sequenced strains were subjected to conjugation experiments in order to investigate the ability of self-transfer of resistance properties to susceptible recipient strains. The 10 donor isolates were mated with one of the two sensitive recipient strains, *E. coli* DH5α (Culture Collection, University of Göteborg, Sweden) and One Shot *E. coli* (Thermo Fisher, USA). Eight of the donor *E. coli* isolates were susceptible to quinolones, and were conjugated with *E. coli* DH5α resistant to nalidixic acid, as recipient. Two of the donor *E. coli* isolates were resistant to quinolones, but susceptible to kanamycin, and were therefore conjugated with One Shot *E. coli* resistant to kanamycin, as recipient. The conjugal transfer was conducted in a Luria-Bertani (LB) broth (Sigma-Aldrich, USA) and the mating was prepared as previously described by Sunde and Sørum (2001). The transconjugant was selected as described by Sunde and Norström (2006), by applying antibacterial disks corresponding to the resistance profile of the donors (Oxoid, UK; Rosco, Denmark) onto the surface of the MH agar plates (BD, USA), with 20 μg/ml nalidixic acid (N-8878 Sigma-Aldrich, USA) or 50 μg/ml kanamycin (K4000 Sigma-Aldrich, USA). The obtained transconjugants were subcultured for inspection of colony morphology as previously described (Sunde and Norström, 2006) and subsequently subjected to susceptibility testing by disk diffusion.

### Multiple-Locus Variable Number Tandem Repeats Analysis

Based on resistance profile, 30 of the 199 isolates were selected for multiple-locus variable number tandem repeats analysis (MLVA). Extraction of DNA was done by dissolving bacterial cells in 350 μl sterile, distilled water (Fresenius Kabi, Germany) and boiling at 100°C for approximately 15 min. Extracted DNA was mixed with reagents from Qiagen Multiplex PCR kit (Qiagen, Germany). The PCR mixture consisted of 12.5 μl of 2x Master mix, 0.5 μl of primer mix and 11 μl of sterile water. Four different primer mixes were used for each DNA sample: EC-5, EC-6, CVN002 and EC-12, where 1 μl extracted DNA was added to the PCR mixtures, to a total volume of 25 μl. The PCR mixtures were placed in the GeneAmp^®^ PCR System 9700 machine (Applied-Biosystems, USA) followed by capillary electrophoresis on an ABI 3130xl Genetic Analyzer (Applied-Biosystems, USA), as described by Løbersli et al. (2012). A control DNA sample (GJ57) was measured along with the unknown DNA samples for quality assurance.

### Molecular Epidemiologic Analysis of the *E. coli* Isolates by BioNumerics

From the MLVA-profiles of the 30 bivalve *E. coli* isolates, the allele numbers generated were entered into BioNumerics database version 7.6 (Applied Maths, Belgium) as character values, and an analysis based minimal spanning tree (MST) clustering was constructed. As markers of genetic relationships, we included 212 community-acquired *E. coli* bacteremia isolates, 38 other human strains from the *E. coli* Reference (ECOR)-collection obtained from the Microbial Evolutionary Laboratory (State University of Michigan, USA), four Enterohemorrhagic *E. coli* (EHEC) strains associated with hemorrhagic uremic syndrome (HUS) from the strain collection at the Norwegian Institute of Public Health, as described (Wester et al., 2013, 2014). The community-acquired *E. coli* isolates causing blood stream infection (BSI) were classified as non-severe, early organ failure (≥organs affected within 1 day of admittance to hospital), or in-hospital death within 14 days of admission (Wester et al., 2013). We applied MST for categorical data, with one-locus difference as first priority rule (weight 10,000), and two-loci difference as second priority rule (weight 10).

## Results

### Sampling and Identification

The majority of the bacterial isolates (90%) were identified as *E. coli*, both by Analytical Profile Index 20E (Oxoid, UK) and by Matrix Assisted Laser Desorption Ionization-Time of Flight Mass Spectrometry (Bruker, Germany). The remaining isolates (10%) belonged to the three genera *Klebsiella, Citrobacter*, and *Enterobacter*.

### Prevalence of Antibacterial Resistance

**FIGURE 1:**
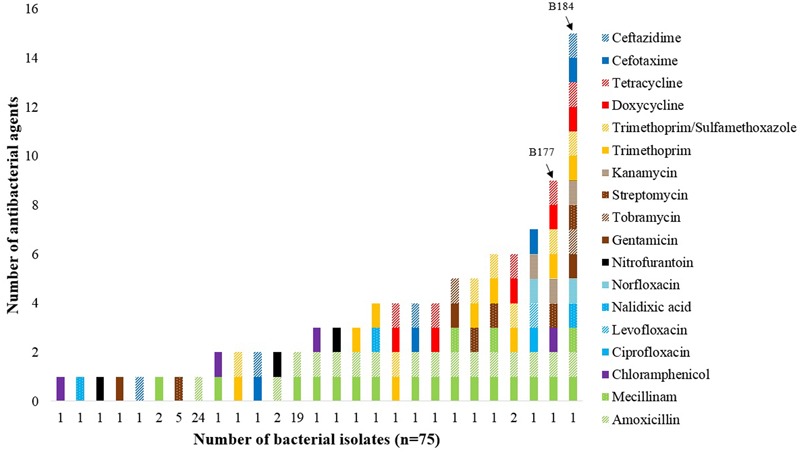
**Number of *E. coli* and other bacteria in the Enterobacteriaceae family showing phenotypic resistance to antibacterial agents applied in accordance with the EUCAST, CLSI, and ICMR clinical breakpoint tables.** The two *E. coli* isolates B177 and B184 (marked by arrows) displayed resistance against nine or more antibacterial agents.

A total of 75 (38%) of the 199 isolates showed resistance to at least one antibacterial agent, while multidrug-resistance was seen in nine (5%) of the isolates (**Figure 1**), using the definition by Magiorakos et al. (2012). Among the 75 resistant isolates, resistance toward extended-spectrum penicillins (83%), aminoglycosides (16%), trimethoprim (13%), sulfonamides (11%), tetracyclines (8%), third-generation cephalosporins (7%), amphenicols (5%), nitrofurans (5%), and quinolones (5%), were observed. Amoxicillin-resistance was found in 59 (79%) isolates, while ampicillin-resistance was found in 36 (48%) isolates. The two *E. coli* isolates B177 and B184 showed phenotypic resistance against nine and 15 antibacterial agents, respectively.

### Genetic Characterization of Selected Resistant *E. coli* Isolates

Among the 10 bacterial isolates subjected to WGS, eight sequence types (STs) were identified. Two isolates belonged to ST-95, and two isolates belonged to ST-58, the remaining six isolates belonged to ST-10, ST-38, ST-69, ST-88, ST-191, or ST-3572, respectively.

Multiple resistance genes were present as examined by ResFinder (**Table 1**). Resistance toward extended-spectrum penicillins was observed in all 10 *E. coli* isolates and they all harbored the *bla*_TEM-1_ gene. Isolate B117 and B184 were resistant to third-generation cephalosporins, and carried the *bla*_CTX-M-15_ and *bla*_CTX-M-14_ genes, respectively. Six isolates possessed genes conferring resistance to aminoglycosides, while five isolates carried genes for resistance against trimethoprim, sulfonamides, and tetracyclines. A gene conferring resistance against amphenicols was observed in two isolates. Two isolates had genes conferring resistance toward quinolones and macrolides, respectively. Notably, three isolates harbored resistance genes (*strA-strB, catA1*, and *qnrS1*, respectively) which did not correspond to the phenotypic resistance pattern.

**Table 1:** Distribution of sequence type (ST), resistance genes, and serotype among 10 *Escherichia coli* isolates by WGS.

Isolate no.	MLST	ResFinder	Serotype
B2	ST-58	*bla*_TEM-1B_, *strA-strB, dfrA5, sul2*	O8:H25
B53	ST-10	*bla*_TEM-1B_	No O type:H4
B117	ST-191	*bla*_TEM-1B_, *bla*_CTX-M-15_	O48:H20
B158	ST-95	*bla*_TEM-1B_, *strA-strB, dfrA5, sul2*	O1:H7
B160	ST-58	*bla*_TEM-1B_, *qnrS1, tet(A)*	O8:H30
B161	ST-69	*bla*_TEM-1B_, *aac(3)-IId*	O17/O44:H18
B165	ST-95	*bla*_TEM-1C_, *strA-strB, dfrA14, sul2, tet(A)*	O1:H7
B167	ST-88	*bla*_TEM-1C_, *tet(A)*	O8:H17
B177	ST-3572	*bla*_TEM-1B_, *strA-strB, dfrA17, sul1, sul2, catA1, aadA5, aph(3′)-Ia, tet(B)*	O89:H9
B184	ST-38	*bla*_TEM-1B_, *bla*_CTX-M-14_, *strA-strB, dfrA17, sul1, sul2, catA1, aac(3)-IId, aadA5, tet(D), mph(A)*	O102:H6

*Genes conferring resistance toward: extended-spectrum penicillins (*bla*_TEM-1_), third-generation cephalosporins (*bla*_CTX-M-14_, *bla*_CTX-M-15_), aminoglycosides [*strA-strB, aadA5, aac(3)-IId, aph(3)-Ia*], trimethoprim (*dfrA17, dfrA5, dfrA14*), sulfonamides (*sul1, sul2*), tetracyclines [*tet(A), tet(B), tet(D)*], amphenicols (*catA1*), quinolones (*qnrS1*), and macrolides (*mphA*).*

### Conjugal Transfer of Antibacterial Resistance Determinants

Six of 10 *E. coli* isolates transferred resistance genes by conjugation (**Table 2**). The three bacterial isolates B2, B158, and B165 transferred trimethoprim- and sulfamethoxazole-resistance, two isolates (B160 and B167) transferred tetracycline-resistance, while one isolate (B117) transferred resistance to cefotaxime and ceftazidime. The resistance patterns of transconjugants were examined by the EUCAST disk diffusion method, in which only a selection of antibacterial agents were employed as determined by the resistance profile of the donor.

**Table 2:** Conjugative transfer and antibacterial resistance (ABR) profile in transconjugants.

Donor	Resistance profile	Conjugation^∗^	Resistance profile transconjugants
B2	AMP-AML-TRI-SXT-S	+	TRI-SUL
B117	AMP-AML-CTX-CAZ	+	AMP-CTX-CAZ
B158	AMP-AML-MEL-TRI-SXT-S	+	TRI-SUL
B160	AMP-AML-TRI-SXT-DO-TE	+	TE
B165	AMP-AML-TRI-SXT-DO-TE	+	TRI-SUL
B167	AMP-AML-DO-TE	+	TE
B53	AMP-AML-NA-TRI	-	
B161	AMP-AML-MEL-GEN-TOB	-	
B177	AMP-AML-C-S-K-TRI-SXT-DO-TE	-	
B184	AMP-AML-MEL-NA-NOR-GEN-TOB-S-K-TRI-SXT-DO-TE-CTX-CAZ	-	

*^∗^Transferability of resistance plasmids; “+” transconjugants were obtained, “-” no transconjugants were obtained. AMP, ampicillin; AML, amoxicillin; MEL, mecillinam; C, chloramphenicol; NA, nalidixic acid; NOR, norfloxacin; GEN, gentamicin; TOB, tobramycin; S, streptomycin; K, kanamycin; TRI, trimethoprim; SUL, sulfamethoxazole; SXT, trimethoprim/sulfamethoxazole; DO, doxycycline; TE, tetracycline; CTX, cefotaxime; CAZ, ceftazidime.*

### Phylogenetic Diversity of the *E. coli* Isolates

**FIGURE 2:**
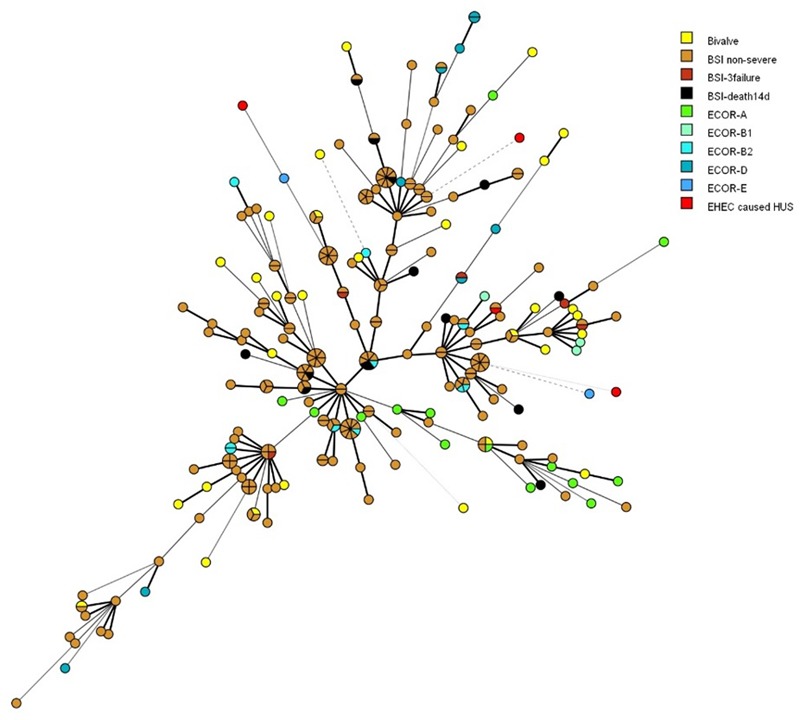
**Minimal spanning tree showing the phylogenetic relationships between 212 *E. coli* isolated from blood stream infection (BSI) according to patient outcome (non-severe, 3failure = organ failure ≥3 organs within one day of admission to hospital, death14d = death within 14 days of admittance to hospital), 38 ECOR strains of human origin with main phylogroup, 30 *E. coli* isolates from bivalves, and four HUS-associated EHEC strains.** The distance between circles are indicated by the thickness and dotting of lines, hence a thicker line indicate a closer relation than a thin line, and a thin line indicate a closer relation than a dotted line. Shared MLVA-profiles are shown as shared circles.

A total of 284 strains were included and MLVA-profiles matching nine specific loci were regarded as phylogenetic related (**Figure 2**). The ECOR strains of different phylogroups and *E. coli* isolates causing BSI did not cluster, nor showed to be located in any specific branch of the MST, except from strains belonging to phylogroup A. The 30 *E. coli* isolates from bivalves seemed to be evenly distributed throughout the MST, together with both the bacteremia *E. coli* and the ECOR strains and the HUS-associated EHEC strains.

## Discussion

Antibacterial resistant fecal bacteria from animals or humans may spread among the human population by direct contact, or via water and food. The transfer of ABR-B in the food production chain may affect the development and spread of resistance among the foodborne pathogens (Sørum and L′Abée-Lund, 2002; VKM, 2015). This could also apply for seafood. Contaminated seafood as fish, bivalves, and crustaceans may cause ABR-B from both marine and fecal origins to reach humans during handling and consumption. A possible risk of transmission of ABR-B may occur from unintentional improper heat treatment, or through bivalves intended for raw- or light preserved consumption. Especially, flat oysters (*Ostrea edulis*) and great scallops (*Pecten maximus*) represents a risk, as they are commonly consumed raw. If these food products are consumed without proper heat treatment, resistant bacteria may enter the consumer and subsequently interact with the intestinal microbiota (Sullivan et al., 2001).

In this study, two *E. coli* isolates displayed phenotypic resistance toward as many as nine or more antibacterial agents, indicating a potential risk of exposure to MDR Enterobacteriaceae during consumption or handling of marine bivalves. In addition, extended spectrum beta-lactamase (ESBL)-producing *E. coli* isolates were identified from this food source (**Table 1**). Among the European countries, Norway has the lowest production corrected use of antimicrobial agents in animals (EMA, 2016). Furthermore, as reported in the *Norwegian monitoring program for antimicrobial resistance in human pathogens, and in bacteria from food, feed and animals* (NORM/NORM-VET, 2015), Norway is a low prevalence country in terms of antimicrobial resistance and it is therefore surprising to detect a high rate of resistant Enterobacteriaceae in marine bivalves, including the ESBL-producing *E. coli* strains. Notably, this should be taken into account in order to determine if bivalves should be included in annual monitoring of ABR in the coastal environment.

The majority of resistant isolates (*n* = 75) examined in the current work were resistant to the extended-spectrum penicillins ampicillin and/or amoxicillin (83%) (**Figure 1**), which is interesting since the use of antimicrobial agents in Norway is dominated by narrow-spectrum penicillins (NORM/NORM-VET, 2015). However, an increase in the use of penicillins with extended spectrum have been reported lately (NORM/NORM-VET, 2015). The increased use of ampicillin and amoxicillin in humans and/or food-producing animals may have led to the development of resistance within the bacterial species observed in this study. Moreover, it is well-known that the *bla*_TEM-1_ gene conferring resistance against extended-spectrum penicillins has been widely distributed in bacterial populations for decades (Hedges et al., 1974). All 10 *E. coli* isolates subjected to in-depth characterization by WGS harbored the *bla*_TEM-1_ gene, whereas two isolates had *bla*_CTX-M_ genes, the latter conferring ESBL-production (**Table 1**). The various TEM enzymes are mutant derivatives of plasmid-mediated beta-lactamases conferring resistance to penicillins, while the CTX-M enzymes confer resistance to penicillins and cephalosporins and have their origin in environmental bacteria (Cantón et al., 2012). The CTX-M enzymes have become the most prevalent ESBLs in bacteria causing human infection, both in hospital and in community settings (Cantón and Coque, 2006; Cantón et al., 2008). The presence of ESBL-positive *E. coli* is of great concern due to possible lack of therapeutic success in the treatment of serious infections, hence defined as critically important by the World Health Organization [WHO] (2014). ESBL-positive *E. coli* have also been recovered from food products for human consumption, as well as from wildlife (Li et al., 2007; Smet et al., 2010; Guenther et al., 2011). A fraction of the bacterial isolates were resistant to aminoglycosides (16%), and six of the 10 sequenced *E. coli* isolates harbored resistance genes. Resistance toward trimethoprim and sulfonamides were seen in 13 and 11% of the isolates, respectively, and five of the 10 sequenced *E. coli* isolates harbored genes conferring resistance toward trimethoprim and sulfonamides. All isolates expressing resistance to trimethoprim and sulfonamides contained genes responsible for the resistance phenotype, except isolates B53 and B160. This indicates that resistance among the bacterial isolates could be a result of selection by increased use, since these agents are synthetic and thus not commonly found in the natural environment. However, observations of resistance toward quinolones and sulfonamides have been seen in the intestinal microbiota of an 11th Century pre-Columbian Andean mummy, showing that resistance even to some synthetic agents may date back to Ancient times (Santiago-Rodriguez et al., 2015).

Among the 10 *E. coli* isolates subjected to conjugation experiments, transferable resistance was detected in six isolates (**Table 2**). The transfer of genes conferring resistance toward third-generation cephalosporins (cefotaxime and ceftazidime) are especially alarming, since the spread of these genes to clinically relevant *E. coli* strains will dramatically reduce the possible choice of antibacterial agents for medical treatment. Moreover, transfer of multiple resistance genes may occur with a higher frequency when the bacteria are exposed to antibacterial agents. ABR among, e.g., enteric bacteria may form reservoirs, in which resistance determinants could transfer to non-resistant bacteria, including those responsible for diseases (Salyers et al., 2004; Stecher et al., 2012). Intestinal bacteria from the human microbiota may, in addition to sharing resistance genes among themselves, also exchange resistance genes to other bacteria that are temporary passing through the intestine (Teuber et al., 1999; Salyers et al., 2004). Thus, commensal bacteria may function as a vector in transferring resistance genes between environmental and pathogenic bacteria.

Whole-genome sequencing and subsequent analysis showed that two isolates belonged to ST-95, while two isolates belonged to ST-38 and to ST-69, respectively (**Table 1**). These STs are associated with bacteremia and urinary tract infection in humans (Adams-Sapper et al., 2012; Alghoribi et al., 2015; Hertz et al., 2016). The MLVA-profiles of the bivalve *E. coli* isolates displayed a seemingly high degree of diversity (**Figure 2**). Furthermore, they scattered among BSI-causing, including those leading to death within 14 days of admission to hospital, as well as among representatives of all *E. coli* main phylogroups. Both instances indicate no common source, but also that the bacteria have the potential for causing serious infection in humans. Consequently, the presence of pathogenic *E. coli* isolates in the coastal environment represent a risk to human health, especially in areas use for aquaculture or recreational activities. This is supported by the findings of Balière et al. (2015) who reported that a few *E. coli* strains of EHEC and Enteropathogenic *E. coli* (EPEC) isolated from bivalve mollusks harbored resistance toward amoxicillin, cefotaxime, and imipenem. The World Health Organization [WHO] (2014) have stated that infections with *E. coli* strains, e.g., EHEC and EPEC, are among the most frequent foodborne causative agents worldwide.

Allochthonous bacteria from different sources (e.g., urban, industrial, and agriculture waste), and residues of antimicrobial agents, will ultimately be transported to the marine environment through waste water eﬄuents, rivers, or streams, and mixed with the indigenous bacterial population (Baquero et al., 2008; Wellington et al., 2013). This can result in the rise of resistance due to selection pressure, and/or genetic exchange between environmental and intestinal bacteria. Bivalves may promote gene transfer among bacteria in the marine environment, by collecting bacteria from various sources and concentrate them within a stable micro-environment at a high density (Taylor et al., 2011). The increasing pressure exerted by antimicrobial agents affects the acquisition, selection, and transmission of resistance determinants among a wide range of bacteria.

## Conclusion

Our study indicates that marine bivalves may represent an important tool for monitoring antibacterial resistant *E. coli* and other members of the Enterobacteriaceae family in coastal environments. Bivalves may furthermore act as a “hot spot” for resistance transfer between Enterobacteriaceae and indigenous bacteria, as the conditions they offer may facilitate the conjugational frequency. As continuous EU programs for the detection of *E. coli* from bivalves are currently implemented, an additional characterization of their ABR profile would represent a good cross-compartment added value indicator of spatial and temporal trends in resistance rates.

## Author Contributions

BL, CS, and DG designed the experimental set up and DG, MS, and AW performed the experiments. DG, CS, MS, AW, and BL wrote the manuscript. All authors agree to be accountable for the content of the work and gave final approval to the manuscript.

## Conflict of Interest Statement

The authors declare that the research was conducted in the absence of any commercial or financial relationships that could be construed as a potential conflict of interest.

## Footnotes

1 https://cge.cbs.dtu.dk/services/
